# Quality, reliability and popularity assessment of Turkish YouTube videos on human papillomavirus and its vaccine: content analysis

**DOI:** 10.7717/peerj.20828

**Published:** 2026-02-18

**Authors:** Hakan Gülmez, Merve Ciftci

**Affiliations:** 1 Department of Family Medicine, Buca Seyfi Demirsoy Training and Research Hospital, Izmir, Turkey; 2 Department of Family Medicine, Faculty of Medicine, Izmir Democracy University, Izmir, Turkey

**Keywords:** YouTube, HPV, HPV vaccine, Human papillomavirus, DISCERN, JAMA, VPI, Health literacy

## Abstract

**Background:**

Human papillomavirus (HPV) is among the most common sexually transmitted viral infections and is associated with significant health burdens, including genital warts and various cancers. YouTube has emerged as a frequently used platform for accessing health-related information, yet the quality and reliability of such content remain uncertain. Individuals seeking information about HPV and its vaccine frequently turn to YouTube, underscoring the need for systematic evaluation of online video content related to HPV vaccination, given its potential to directly influence public engagement and vaccine acceptance. Analyzing YouTube videos provides insight into the level of information users are exposed to, the potential risks of misinformation, and the overall reliability of health-related digital content. Such evaluations contribute to strengthening public health strategies and support efforts to enhance access to accurate and trustworthy information.

**Objective:**

This study aimed to evaluate the information quality, reliability, and popularity of Turkish-language YouTube videos related to HPV and the HPV vaccine.

**Methods:**

This cross-sectional study was conducted on the YouTube platform using the keywords ‘HPV,’ ‘HPV vaccine (“HPV aşısı”),’ and ‘human papillomavirus,’ and the videos were viewed between July 1 and 31, 2025. The first 200 videos for each keyword were screened, and those meeting the inclusion criteria were analyzed. Video characteristics (*e.g.*, duration, views, likes, comments, like ratio) and content features (*e.g.*, type, source, presentation format, narrator, purpose, citation of sources, recency, vaccine recommendations, anti-vaccine stance) were recorded. Information quality was assessed using the DISCERN instrument (16–80), reliability using the Journal of the American Medical Association (JAMA) Benchmark criteria (0–4), and popularity using the Video Power Index (VPI). Statistical analyses included descriptive statistics, *t*-tests, ANOVA, chi-square tests, and Pearson correlation analysis, with *P* < .05 considered significant.

**Results:**

A total of 600 videos were analyzed, with 270 of them meeting the inclusion criteria. The mean duration was 5.62 ± 13.98 minutes (range: 0.12–147), and the mean number of views was 35,829.5 ± 156,796.8 (range: 131–2,260,592). The mean DISCERN, JAMA, and VPI scores of the videos were calculated as 47.6 ± 15.1 (range: 16–80), 1.9 ± 0.9 (range: 0–4), and 82.9 ± 318.6 (range: 0–3,980), respectively. More than half of the videoswere prepared by healthcare professionals (52.9%, *n* = 143). When categorized by content, a relatively large proportion of the videos focused on vaccination (40.4%, *n* = 109). A substantial proportion of the videos (58.5%, *n* = 158) explicitly recommended the HPV vaccine, while only a small proportion (1.9%, *n* = 5) expressed an anti-vaccine stance. As videos are presented by healthcare professionals and their duration increases, the quality of information improves; however, this quality does not appear to be directly associated with popularity.

**Conclusions:**

Currently, YouTube has become a frequently utilized platform for sharing health information. However, a significant portion of the analyzed HPV-related content is inadequate in terms of information quality and reliability. Promoting longer-duration, current and evidence-based videos prepared by healthcare professionals that cite reliable sources may contribute to the improvement of digital health literacy in the general population.

## Introduction

Human papillomavirus (HPV) is one of the most common sexually transmitted viral infections, causing not only genital warts in both women and men but also serious clinical conditions such as cervical, anal, vaginal, vulvar, penile, and oropharyngeal cancers (Mavundza et al., 2021). It is known that the vast majority of sexually active individuals will be infected with HPV at least once in their lifetime (Giuliani et al., 2020).

The World Health Organization has stated that cervical cancer is the first type of cancer that could be eliminated on a global scale over time and, in 2020, launched the Cervical Cancer Elimination Initiative (Bakanlığı  et al. , 2021). As part of this program, all countries have been recommended to implement HPV vaccination and appropriate screening strategies, aiming to combat cervical cancer not only at the individual level but also globally. Health literacy and access to accurate information are critical to the success of this goal.

Today, the internet has become one of the most frequently used sources for accessing health information, as in many other fields (Global Media Insight, 2025; Mendi, 2015). Due to its accessibility and cost-free nature, YouTube has emerged as a preferred video-sharing platform for individuals seeking health-related information (Madathil et al., 2015). The literature contains numerous studies evaluating the content and quality of YouTube videos on various medical topics as sources of information, and research in this field is continuously expanding (Azer, 2020; Büyüktopçu & Kart, 2025; Jildeh et al., 2021; Keelan et al., 2007; Li et al., 2020; Yazla et al., 2024).

In this context, individuals seeking information about HPV and the HPV vaccine also frequently turn to YouTube. The quality and reliability of digital information sources are critically important, particularly for public health topics such as the HPV vaccine. Indeed, the 2025 report of the National Academies of Sciences, Engineering, and Medicine underscores that although access to scientific information on digital platforms is steadily increasing, the transparency, accuracy, and source reliability of such content remain highly variable—an issue that constitutes a key factor driving the risk of misinformation (National Academies of Sciences, Engineering, and Medicine, 2025). Therefore, there is a need for the systematic evaluation of online content related to HPV and the HPV vaccine, given its potential influence on vaccine acceptance. Analyzing YouTube videos provides critical insights into the level of information users encounter, the potential risks of misinformation, and the overall reliability of online health information, thereby contributing both to the strengthening of public health policies and to supporting access to accurate and trustworthy information.

Various studies on HPV and HPV vaccination exist in the current literature (Ache & Wallace, 2008; Briones et al., 2012; Di Spirito et al., 2023; Ekram et al., 2019; Kim et al., 2021). Although there is a Turkish-language study on penile cancer that one of the HPV-associated cancers, which focuses solely on penile cancer awareness and does not encompass general information on HPV or its vaccine (Günay, Gelmiş & Dizdaroğlu, 2025). Therefore, there is currently no study that provides a comprehensive evaluation of Turkish digital content related to HPV and the HPV vaccine.

Moreover, the fact that the HPV vaccine has not yet been integrated into the national immunization program in Turkey further underscores the need to effectively disseminate accurate and reliable information at the community level and to develop strategies aimed at increasing vaccine acceptance. Therefore, the aim of this study was to evaluate the information quality, reliability, and popularity of Turkish-language YouTube videos related to HPV and the HPV vaccine.

## Materials & Methods

### Study design

This cross-sectional study was conducted on the YouTube platform using the keywords ‘HPV,’ ‘HPV vaccine (“HPV aşısı”),’ and ‘human papillomavirus.’ The videos were viewed between July 1 and 31, 2025, and the viewing dates and YouTube posting dates were systematically recorded. Search results were sorted by view count, and the first 200 videos for each keyword were selected, yielding a total of 600 videos for preliminary screening. All searches were performed in Google Chrome’s incognito mode, with browsing history cleared before each search. Inclusion criteria required videos to be in Turkish, contain health-related information, and be directly relevant to HPV or the HPV vaccine. Duplicate videos, non-Turkish videos, videos unrelated to HPV or its vaccine, those with insufficient or poor audio quality, and videos with the “like” function disabled were excluded. Based on these criteria, 270 videos were included for analysis. The videos were independently analyzed by two raters (MC and HG). Any discrepancies between the assessments were resolved through repeated discussions and mutual consensus, thereby ensuring the reliability and consistency of the process. This approach has also been employed in similar content analysis studies on other diseases (Polat & Cankurtaran, 2025).

### Data review

For each video, the following data were recorded: title, URL, upload date, duration, number of views, total number of comments, total number of likes, like ratio, channel name, number of channel subscribers, channel type, time elapsed since upload, and the date of evaluation. Video duration groups were classified according to YouTube’s search filter categories (<4 min, 4–20 min, and >20 min) along with the “YouTube Shorts” format for videos shorter than 1 min.

Videos were evaluated based on several criteria, including content type, source, presentation format, narrator type, purpose, citation of information sources, recency of information, recommendations for vaccination, and anti-vaccine stance.

### Video popularity

In our study, we utilized the Video Power Index (VPI), a metric developed by Erdem & Karaca (2018) and widely employed in previous research, to indicate the popularity level of videos. The like ratio was calculated as (number of likes ×100)/(number of likes + number of dislikes), the view rate as (number of views/number of days since upload), and the VPI value, used to assess video popularity, as (like ratio × view rate)/100 (Kuru & Erken, 2020). As YouTube removed the public display of dislike counts for all videos as of November 2021, the total number of dislikes could not be recorded, and this value was assumed to be 0 in the related formula calculations (The YouTube Team, 2021).

### Quality and reliability evaluation

The quality and reliability of the videos were evaluated using the DISCERN instrument and the Journal of the American Medical Association (JAMA) Benchmark criteria. DISCERN is a 16-item assessment tool developed by the University of Oxford to evaluate the quality of health-related written and visual materials (Charnock et al., 1999). The questions are organized into three main domains: content reliability, adequacy of treatment options, and overall quality. Each item is rated on a scale from 1 (very poor) to 5 (excellent), yielding a total score ranging from 16 to 80. Based on the total score, quality is classified into five categories: very poor (<26), poor (27–38), fair (39–50), good (51–62), and excellent (>63). The JAMA Benchmark is a four-item international criterion used to assess the reliability of health-related content on the internet (Silberg, Lundberg & Musacchio, 1997). First proposed in 1997 by the JAMA, it evaluates four key elements: (1) authorship, (2) attribution (references, copyright information), (3) disclosure (sponsorship, advertising, conflicts of interest), and (4) currency (indication of the dates that content was posted and updated). Each criterion is scored as 1 if present or 0 if absent, resulting in a total score ranging from 0 to 4. A score of ≥3 is considered “high reliability,” while a score of ≤2 indicates “low reliability.”

While the JAMA Benchmark highlights the transparency and structural reliability of the source, DISCERN assesses the quality and evidence-based nature of treatment-related information. The distinct yet complementary focuses of these two tools create a robust evaluative framework that enables the systematic and holistic assessment of online health content. The combined use of the JAMA Benchmark and the DISCERN instrument provides a comprehensive and balanced approach to evaluating online health information.

### Statistical analyses

The statistical analyses were performed using IBM SPSS Statistics for Windows, Version 22.0 (IBM Corp., Armonk, NY, USA). Descriptive statistics were calculated as mean ± standard deviation (SD), minimum, and maximum values for numerical variables, and as frequency (n) and percentage (%) for categorical variables. The assumption of normality was assessed using the Kolmogorov–Smirnov test, and parametric tests were applied for variables that followed a normal distribution. For group comparisons, the independent samples *t*-test was used for two-group analyses, and one-way analysis of variance (ANOVA) was used for comparisons involving more than two groups. The Pearson chi-square (*χ*^2^) test was employed to examine associations between categorical variables. Pearson correlation analysis was conducted to examine linear relationships between variables. A *P*-value of <.05 was considered statistically significant for all analyses.

### Ethical considerations

The study was conducted in accordance with the ethical standards outlined in the Declaration of Helsinki and relevant national regulations. As no human or animal data were used, ethics committee approval was not required. The analysis was based on publicly available YouTube videos. No identifiable personal data were used, and all results are presented in aggregate form; therefore, formal ethical approval was deemed unnecessary.

## Results

For each keyword, the first 200 videos listed in the search results were selected, yielding a total of 600 videos for preliminary assessment. Based on the predefined exclusion criteria, 330 videos were excluded from the study. Of these, 244 were in languages other than Turkish, 19 contained promotional content, two had inadequate audio quality, and 65 were duplicate videos. Consequently, 270 videos meeting the inclusion criteria were subjected to detailed analysis.

In the keyword-based evaluation, the search term “HPV vaccine (“HPV aşısı”)” yielded the highest inclusion rate (*n* = 132) and the lowest proportion of non-Turkish content (*n* = 2). In contrast, the search term “human papillomavirus” had a low inclusion rate (*n* = 40) due to the high proportion of foreign language videos (*n* = 149). Furthermore, the majority of duplicate videos (*n* = 46) and promotional content (*n* = 19) were found in the results for the keyword “HPV aşısı.”

The mean duration of the videos was 5.62 ± 13.98 min (range: 0.12–147). The videos had a mean view count of 35,829.5 ± 156,796.8 (range: 131–2,260,592). The mean time elapsed since upload was 988.9 ± 994.1 days (range: 0–5,423), and the mean view rate was 83.9 ± 318.5 (range: 0–3,980). The channels had a mean subscriber count of 142,549.7 ± 261,649.7 (range: 7–1,330,000). On average, the videos received 431.1 ± 3,039.3 likes (range: 0–49,000) and 49.5 ± 152.9 comments (range: 0–1,444). The mean like ratio was 95.6 ± 20.7 (range: 0–100). The mean DISCERN, JAMA, and VPI scores of the videos were calculated as 47.6 ± 15.1 (range: 16–80), 1.9 ± 0.9 (range: 0–4), and 82.9 ± 318.6 (range: 0–3,980), respectively.

The overall distribution of the other criteria evaluated in the videos is presented in Table 1.

**Table 1:** Distribution of general findings related to the content of the videos.

		n	%
YouTube Channel Type	News-TV Channel	44	16.3
	Private Hospital	47	17.4
	Private YouTube Channel	32	11.8
	Official Institution	4	1.4
	Healthcare Professional	143	52.9
Video Content	Vaccine	109	40.4
	Signs and Symptoms	8	3.0
	Transmission	14	5.2
	General Information	93	34.4
	Patient Experience	1	0.4
	Rehabilitation	3	1.1
	Screening	9	3.3
	Treatment and Prevention	33	12.2
Presentation Style	Animation	16	5.9
	News/Interview	66	24.4
	Patient Experience	26	9.6
	Expert Commentary	162	60.0
Narrator	Unidentified narrator	28	10.4
	Physician	229	84.8
	Pharmacist	1	0.4
	Patient	11	4.1
	None	1	0.4
Purpose	Informative	233	86.3
	Sharing of Experience	6	2.2
	Promotion	10	3.7
	Discussion	8	3.0
	Inducement	13	4.8
Is the source of information specified?	Yes	13	4.8
	No	257	95.2
Is the information up to date?	Yes	209	77.4
	No	61	22.6
Is the HPV vaccine explicitly recommended in the video?	Yes	158	58.5
	No	112	41.5
Is the HPV vaccine hesitancy or opposition reflected in the video?	Yes	5	1.9
	No	265	98.1
Total		**270**	**100**

In the evaluation based on the source of the videos, statistically significant differences were observed in DISCERN and JAMA scores (*P* < 0.05 and *P* < 0.001, respectively). However, no significant association was found between VPI scores and the video source (*P* = 0.11).

When videos produced by private hospitals and healthcare professionals were compared, those from private hospitals had higher DISCERN and VPI scores (51.8 ± 14.3 and 84.2 ± 394.03, respectively); however, these differences were not statistically significant (*P* = 0.244 and *P* = 0.226, respectively). Although JAMA scores were also higher for private hospital videos, this difference was not statistically significant (Table 2; *P* = 0.058).

**Table 2:** Mean DISCERN, JAMA, and VPI scores by video source.

		**n**	**Mean**	**SD** ^d^	**Min.** ^e^	**Max.** ^f^	***P*** ** value**
DISCERN^a^	News-TV Channel	44	42.4	15.9	16	76	0.009^*^
	Private Hospital	47	51.8	14.3	19	80	
	Private YouTube Channel	32	42.9	15.2	16	76	
	Official Institution	4	45	9.1	32	52	
	Healthcare Professional	143	48.9	14.7	16	80	
	Total	270	47.6	15.1	16	80	
JAMA^b^	News-TV Channel	44	1.5	1.1	0	4	0.000^**^
	Private Hospital	47	2.2	0.7	0	4	
	Private YouTube Channel	32	1.6	0.9	0	4	
	Official Institution	4	2.5	1	2	4	
	Healthcare Professional	143	1.9	0.7	0	4	
	Total	270	1.9	0.9	0	4	
VPI^c^	News-TV Channel	44	157.1	257.7	0	1,065	0.111
	Private Hospital	47	84.2	394.03	0	2,716	
	Private YouTube Channel	32	172.4	700.7	1	3,980	
	Official Institution	4	36.5	40.9	1	72	
	Healthcare Professional	143	40.9	96.5	0	717	
	Total	270	82.9	318.6	0	3,980	

**Notes.**

a DISCERN, Quality Criteria for Consumer Health.

b JAMA, Journal of the American Medical Association.

c VPI, Video Power Index.

d SD, Standard Deviation.

e Min., Minimum.

f Max., Maximum,

* *P* < 0.05.

** *P* < 0.001.

In addition, the mean VPI scores of videos published by private YouTube channels and News-TV channels (172.4 ± 700.7 and 157.1 ± 257.7, respectively) were found to be higher compared to other sources; however, this difference was also not statistically significant (*P* = 0.111).

The mean DISCERN, JAMA, and VPI scores according to the video source are presented in Table 2.

When evaluated according to video content, statistically significant differences were found in DISCERN and JAMA scores (*P* = 0.001 and *P* = 0.005, respectively). In contrast, no significant association was observed between VPI scores and content type (*P* = 0.976).

A substantial proportion of the content addressed the topic of vaccine (40.4%, *n* = 109). Despite their high VPI scores (102.42 ± 408.7), these videos were notable for having low DISCERN and JAMA scores (43.2 ± 15.3 and 1.6 ± 0.9, respectively). On the other hand, videos focusing on patient experiences were found to be exceedingly rare (0.4%, *n* = 1) and lacked sufficient representational power for a meaningful analysis of score distribution.

The mean DISCERN, JAMA, and VPI scores according to video content are presented in Table 3.

**Table 3:** Mean DISCERN, JAMA, and VPI scores by video content.

		**n**	**Mean**	**SD** ^d^	**Min.** ^e^	**Max.** ^f^	***P*** ** value**
DISCERN^a^	Vaccine	109	43.2	15.3	16	80	0.001^*^
	Signs and Symptoms	8	45	9.2	34	62	
	Transmission	14	49.1	11.03	36	72	
	General Information	93	51.3	14.8	16	77	
	Patient Experience	1	39	–	39	39	
	Rehabilitation	3	40.7	24.5	16	65	
	Screening	9	43	15.1	16	69	
	Treatment and Prevention	33	54.2	13.2	26	78	
	Total	270	47.6	15.1	16	80	
JAMA^b^	Vaccine	109	1.6	0.9	0	4	0.005^*^
	Signs and Symptoms	8	1.9	0.35	1	2	
	Transmission	14	1.9	0.3	1	2	
	General Information	93	2.1	0.7	0	4	
	Patient Experience	1	1	–	1	1	
	Rehabilitation	3	1.3	1.2	0	2	
	Screening	9	1.8	0.7	0	2	
	Treatment and Prevention	33	2.1	0.7	0	4	
	Total	270	1.9	0.9	0	4	
VPI^c^	Vaccine	109	102.4	408.7	0	3,980	0.976
	Signs and Symptoms	8	36.8	59.7	2	180	
	Transmission	14	28.9	52.2	2	185	
	General Information	93	88.4	311.3	0	2,716	
	Patient Experience	1	161	–	161	161	
	Rehabilitation	3	86.3	89.9	17	188	
	Screening	9	32.9	50.2	2	164	
	Treatment and Prevention	33	47.9	48.5	0	157	
	Total	270	82.9	318.6	0	3,980	

**Notes.**

a DISCERN, Quality Criteria for Consumer Health.

b JAMA, Journal of the American Medical Association.

c VPI, Video Power Index.

d SD, Standard Deviation.

e Min., Minimum.

f Max., Maximum.

* *P* < 0.05.

When evaluated by duration, 46.3% (*n* = 125) of the analyzed videos were shorter than 1 min, 28.1% (*n* = 76) were between 1 and 4 min, 20% (*n* = 54) were between 4 and 20 min, and 5.6% (*n* = 15) were longer than 20 min. Based on this distribution, nearly half of the videos consisted of content classified as YouTube Shorts, which are under 1 min in length.

In the analysis by duration groups, DISCERN and JAMA scores were found to increase significantly as the video duration increased (*P* < 0.001 for both). Videos shorter than 1 min (46.3%; *n* = 125) had the lowest DISCERN and JAMA scores (40.2 ± 11.2 and 1.6 ± 0.8, respectively), whereas those longer than 20 min (5.6%; *n* = 15) attained the highest DISCERN and JAMA scores (65.1 ± 16.4 and 2.8 ± 1.2, respectively).

In contrast, no statistically significant difference was found between the groups in terms of VPI scores (*P* = 0.267). The mean VPI scores were calculated as follows: 124.3 ± 450.8 for videos shorter than 1 min, 51.6 ± 139.7 for videos between 1–4 min, 43.9 ± 58.2 for videos between 4–20 min, and 36.9 ± 63.3 for videos longer than 20 min.

The mean DISCERN, JAMA, and VPI scores according to video duration are presented in Table 4.

**Table 4:** Mean DISCERN, JAMA, and VPI scores by video duration.

		**n**	**Mean**	**SD** ^d^	**Min.** ^e^	**Max.** ^f^	***P*** ** value**
DISCERN^a^	Videos shorter than 1 min	125	40.2	11.2	16	67	0.000^*^
	Videos between 1–4 min	76	47.9	12.3	19	75	
	Videos between 4–20 min	54	59.5	14.8	22	80	
	Videos longer than 20 min	15	65.1	16.4	31	80	
	Total	270	47.6	15.1	16	80	
JAMA^b^	Videos shorter than 1 min	125	1.6	0.8	0	4	0.000^*^
	Videos between 1–4 min	76	1.8	0.5	0	4	
	Videos between 4–20 min	54	2.2	0.9	0	4	
	Videos longer than 20 min	15	2.8	1.2	1	4	
	Total	270	1.9	0.9	0	4	
VPI^c^	Videos shorter than 1 min	125	124.3	450.8	0	3,980	0.267
	Videos between 1–4 min	76	51.6	139.7	0	717	
	Videos between 4–20 min	54	43.9	58.2	0	225	
	Videos longer than 20 min	15	36.9	63.3	1	245	
	Total	270	82.9	318.6	0	3,980	

**Notes.**

a DISCERN, Quality Criteria for Consumer Health.

b JAMA, Journal of the American Medical Association.

c VPI, Video Power Index.

d SD, Standard Deviation

e Min., Minimum

f Max., Maximum

* *P* < 0.001.

When evaluated according to narrator type and content, the vast majority of videos (84.8%; *n* = 229) were narrated by physicians, and the most frequently addressed topic was the vaccine (40.4%; *n* = 109). Most vaccine-related videos were narrated by physicians (35.8%; *n* = 82) and by individuals with an unidentified narrator identity (17.4%; *n* = 19). Additionally, 63.6% (*n* = 7) of the videos narrated by patients were also related to the vaccine.

Of the videos, 58.5% (*n* = 158) explicitly recommended the HPV vaccine, while only 1.9% (*n* = 5) expressed an anti-vaccine stance. The remaining 39.6% (*n* = 107) contained neither recommendations for nor opposition to the vaccine. Content recommending the vaccine was most frequently found in videos narrated by physicians (58.1%; *n* = 133) and patients (72.7%; *n* = 8).

When comparing information quality levels by video source, no statistically significant association was found between video source and DISCERN score categories (*χ*^2^ (20, *n* = 270) = 26.62; *P* = 0.15). A substantial proportion of videos uploaded by private hospitals (27.7%; *n* = 13) provided content rated as “excellent,” while healthcare professional channels similarly featured a high proportion of “excellent” (25.9%; *n* = 37) and “fair” (32.2%; *n* = 46) quality content. In contrast, News-TV channels had higher proportions of both “very poor” (13.6%; *n* = 6) and “poor” (36.4%; *n* = 16) content. Although videos produced by private hospitals and healthcare professionals contained a higher proportion of “excellent” quality content, this difference was not statistically significant. Furthermore, the low number of videos from official institutions limited the representativeness of this group in the analysis.

When information quality was evaluated by video duration, content quality was found to increase markedly as the video duration increased (*χ*^2^ (12, *n* = 270) = 116.19; *P* < 0.001). Notably, 80% (*n* = 12) of videos longer than 20 min achieved DISCERN scores corresponding to the “excellent” category, representing the highest content quality among all groups. Similarly, 59.3% (*n* = 32) of videos lasting 4–20 min were rated as “excellent,” and 20.4% (*n* = 11) as “good.” In contrast, only 2.4% (*n* = 3) of videos shorter than 1 min provided “excellent” quality content, while 82.4% (*n* = 103) were rated as “very poor,” “poor,” or “fair.”

The distribution of quality according to DISCERN score categories by YouTube channel type and video duration is presented in Table 5.

**Table 5:** Distribution of quality according to DISCERN score groups by YouTube channel type and video duration.

			**DISCERN** ^a^ ** Score Groups**					**Total**	*χ* ^2^ ^b^	**df** ^c^	***P*** ** value**
			**Very poor**	**Poor**	**Fair**	**Good**	**Excellent**				
Channel Type	News-TV Channel	n	6	16	10	6	6	44	26.62	20	0.15
		%	13.6	36.4	22.7	13.6	13.6	100			
	Private Hospital	n	1	9	13	11	13	47			
		%	2.1	19.1	27.7	23.4	27.7	100			
	Private YouTube Channel	n	5	8	11	3	5	32			
		%	15.6	25	34.4	9.4	15.6	100			
	Official Institution	n	0	1	2	0	1	4			
		%	0	25	50	0	25	100			
	Healthcare Professional	n	11	25	46	24	37	143			
		%	7.7	17.5	32.2	16.8	25.9	100			
	Total	n	23	59	82	45	61	270			
		%	8.5	21.9	30.4	16.7	22.6	100			
Duration Group	Videos shorter than 1 min	n	16	38	49	19	3	125	116.19	12	0.000^*^
		%	12.8	30.4	39.2	15.2	2.4	100			
	Videos between 1–4 min	n	3	15	29	15	14	76			
		%	3.9	19.7	38.2	19.7	18.4	100			
	Videos between 4–20 min	n	4	3	4	11	32	54			
		%	7.4	5.6	7.4	20.4	59.3	100			
	Videos longer than 20 min	n	0	3	0	0	12	15			
		%	0	20	0	0	80	100			
	Total	n	23	59	82	45	61	270			
		%	8.5	21.9	30.4	16.7	22.6	100			

**Notes.**

a DISCERN, Quality Criteria for Consumer Health.

b *χ*^2^, Pearson Chi-Square.

c df, degrees of freedom.

* *P* < 0.001.

In the correlation analysis based on video upload dates, no significant associations were found between upload date and either DISCERN scores (*r* = −0.03, *P* = 0.59) or JAMA scores (*r* = −0.07, *P* = 0.22).

## Discussion

This study evaluated the information quality, reliability, and popularity of Turkish-language YouTube videos related to HPV and the HPV vaccine, and examined the influence of factors such as video duration, source, content theme, and narrator type on these outcomes. The findings indicate that video quality is largely dependent on content, duration, and presentation characteristics, whereas popularity (VPI) does not appear to be directly associated with information quality.

During the data collection process, the evaluation by keywords revealed that the search term “HPV aşısı” (“HPV vaccine”) had the highest inclusion rate and the lowest proportion of foreign language content, indicating that keyword selection significantly influences the quality of retrieved content.

In our study, more than half of the evaluated videos (52.9%) were presented by healthcare professionals, the majority of whom were physicians (84.8%). This finding underscores the significant role of healthcare professionals in disseminating information about HPV on digital platforms. The literature similarly reports that videos produced by healthcare professionals achieve higher scores in terms of information quality and reliability compared to other sources, a finding consistent with the results of our study (Büyüktopçu & Kart, 2025; Di Spirito et al., 2023; Jildeh et al., 2021; Kaşıkcı& Yıldırım, 2021; Li et al., 2020). Furthermore, in the study by Demircan, Kıyak & Kaya (2024), which evaluated videos on heparin injection techniques, it was reported that content produced by healthcare institutions and official organizations achieved higher quality scores. In addition, videos uploaded by healthcare facilities or hospitals had significantly higher view counts compared to those uploaded by individual users. In contrast, in our study, when videos produced by private hospitals were compared with those created by healthcare professionals, the former had higher DISCERN and VPI scores; however, these differences were not statistically significant (*P* = 0.244 and *P* = 0.226, respectively).

In our study, the overall information quality of the evaluated YouTube videos was found to be generally low, although certain factors appeared to influence the quality level. This finding is consistent with previous research, which has also reported that a substantial proportion of health-related YouTube content possesses moderate or low information quality. Analysis based on DISCERN score categories revealed that only 22.6% of the videos fell into the “excellent” category, confirming, as reported by Aydin & Akyol (2020) in thyroid cancer videos and D’Ambrosi et al. (2025) in videos on the anterolateral ligament of the knee, that high-quality content remains in the minority on the YouTube platform. Similarly, in the study by Di Spirito et al. (2023) examining videos on HPV-related oral lesions, the vast majority of videos were reported to be categorized as having “moderate” or lower levels of information quality. In contrast, the literature also includes studies demonstrating a predominance of high-information-quality videos, as reported by Yavuz, Buyuk & Genc (2020) in their evaluation of YouTube videos on “accelerated orthodontics”.

On the other hand, our study found that content quality increased markedly with video length (*χ*^2^(12, *n* = 270) = 116.19; *P* < 0.001). In particular, videos longer than 20 min achieved the highest mean DISCERN and JAMA scores, with 80% rated as “excellent.” This finding suggests that short videos are insufficient for informative purposes, whereas longer videos provide more comprehensive and reliable information. Moreover, this result is consistent with the findings of Demircan, Kıyak & Kaya (2024); Öztürk et al. (2021), who reported that longer and more detailed educational content is associated with higher quality scores. Similarly, in the study conducted by Ha, Hur & Mun (2023) on wart-related content on YouTube, it was demonstrated, consistent with our findings, that the quality and reliability of videos were closely associated with both the type of narrator and the duration of the video. Videos prepared by healthcare professionals were shown not only to provide higher-quality information but also, owing to their longer duration, to offer users more comprehensive and evidence-based knowledge. However, in contrast to our findings, there are studies in the literature reporting no significant association between video duration and quality scores. For example, in their evaluation of YouTube videos on inhaler use, Canbolat et al. (2024) found negative correlations between video quality (Global Quality Score –GQS) and the number of views, likes, comments, as well as video duration.

In our study, analysis of video content distribution revealed that the most frequently addressed topic was the HPV vaccine (40.4%). Although vaccine-related videos had relatively high VPI scores, their information quality and reliability levels were lower compared to other content types, indicating that popularity does not always align with content quality. This finding is consistent with observations frequently emphasized in the literature, which highlight that highly viewed content on social media platforms may have limitations in terms of information quality (Osman et al., 2022).

In contrast, videos focusing on “treatment and prevention” demonstrated significantly higher information quality (DISCERN:54.2 ± 13.2) and reliability scores (JAMA:2.1 ± 0.7) compared to other topics (*P* = 0.001 and *P* = 0.005, respectively). This finding is consistent with previous studies reporting that health education and clinically informative content are associated with higher quality scores. For example, in their evaluation of YouTube videos on chemotherapy using the modified DISCERN (mDISCERN) and other quality metrics, (Sahin & Seyyar, 2023) reported that patient-oriented informational videos generally achieved higher quality scores.

Although numerous studies in the literature have reported that negative content regarding the HPV vaccine is predominant, the present study found that a significant portion of videos (58.5%) explicitly recommended vaccination, with only 1.9% containing anti-vaccine content. For instance, Ekram et al. (2019); Keelan et al. (2007) reported that a considerable proportion of HPV vaccine–related videos on YouTube contained misinformation or negative discourse, particularly emphasizing potential adverse effects. Similarly, Briones et al. (2012) reported in their study that the vast majority of videos had a negative tone, did not endorse the HPV vaccine, and that such negative content received more likes from viewers compared to positive or neutral videos. In contrast, and consistent with the findings of our study, research conducted by Ache & Wallace (2008), as well as by Covolo et al. (2017), indicated that the majority of YouTube videos emphasized the benefits of the vaccine, with negative content being relatively limited.

On the other hand, a study conducted in Italy on measles-mumps-rubella (MMR) vaccination among children suggested a possible association between the decline in vaccination rates and the presence of misinformation disseminated in online environments (Donzelli et al., 2018). In the study by Tang et al. (2021) on the recommendation algorithm of anti-vaccine videos on YouTube, it was reported that social media platforms such as YouTube facilitate the spread of misinformation about vaccines, and that anti-vaccine videos tend to have higher view counts. Similarly, numerous studies have found that anti-vaccination videos have higher view rates; accordingly, it has been emphasized that healthcare professionals should develop strategies to reduce misinformation on online platforms and ensure that users are made aware of such content (Briones et al., 2012).

In addition, in the study conducted by Elkin, Pullon & Stubbe (2020), vaccine-related content on Google, Facebook, and YouTube was comparatively evaluated. The findings indicated that approximately 80% of the websites on Google and 75% of the videos reviewed on YouTube were supportive of vaccination, whereas around 50% of the content shared on Facebook contained anti-vaccination views. These results highlight the importance of comparatively examining vaccine-related information and attitudes across different social media platforms (Elkin, Pullon & Stubbe, 2020).

A study based on Google Trends data reported a significant upward trend in online searches related to the HPV vaccine between 2010 and 2021 (Bhagavathula & Massey, 2022). The analysis revealed an average annual increase of 8.6% during this period and demonstrated that major changes in national vaccination policies and official recommendations led to noticeable surges in search volumes. Similarly, in the study by Southwell et al. (2016) examining online information-seeking and social media engagement related to the Zika virus, sudden increases in news coverage were shown to produce pronounced but short-lived spikes in Google search activity and Twitter posts. The study demonstrated that official statements issued by national health authorities, particularly the World Health Organization, were strongly associated with increases observed in online search behavior (*e.g.*, *r* = 0.86 for the United States). Taken together, these findings indicate that online search trends serve as an important indicator of public awareness and interest in critical public health issues such as the HPV vaccine, while also suggesting that official communications and health policies exert a significant influence on information-seeking behavior.

In our study, the notably high rate of vaccination recommendations in videos presented by physicians (58.1%) underscores the significant role of healthcare professionals in educating the public through digital platforms and their critical impact on promoting positive attitudes toward HPV vaccination. This finding is also consistent with the results of the study by Margolis et al. (2022), which examined health care providers’ approaches following HPV vaccine refusal. Their study demonstrated that post-visit follow-up practices significantly increased the likelihood of subsequent vaccine acceptance among parents (43% among those who received follow-up *vs.* 20% among those who did not). The study also underscored the pivotal role of health care professionals in guiding and informing parents throughout the decision-making process.

It has been reported in a content analysis of YouTube videos on the HPV vaccine in Japan that anti-vaccine videos increased in 2013, whereas the majority of those uploaded since 2019 have conveyed positive attitudes toward vaccination (Xu et al., 2024). In this context, as emphasized by Brandt, Pierce & Crary (2016), it is recommended to increase the number of evidence-based, high-quality contents that advocate HPV vaccination to improve public health, as well as to support such content through public health policies and official institutions.

A web-based pilot study investigating misinformation dynamics related to PrEP and HIV vaccines in sub-Saharan Africa provides indirect support for the critical role of health professionals in digital environments (Marton et al., 2021). Survey findings indicated that misinformation was propagated largely through friends and family, close social networks, and particularly *via* social media platforms such as WhatsApp. Given the difficulty of formally monitoring these channels, the study highlights the need for additional research to better understand the mechanisms through which misinformation spreads and to develop effective intervention strategies. The findings further underscore that relying solely on official information sources is insufficient; instead, multi-layered communication approaches that center on community structures and social network dynamics are required.

In summary, our study determined that HPV-related videos on YouTube generally exhibit moderate information quality and low source reliability. No direct relationship was observed between video popularity and content quality; notably, private YouTube channels demonstrated higher VPI scores but lower DISCERN scores. These findings align with systematic reviews evaluating general YouTube content analyses. For instance, in the study by Osman et al. (2022) which assessed a total of 22,300 videos from 202 articles published up to August 2020, it was reported that health-related content on YouTube was predominantly of average or below-average quality, with no significant correlation between popularity and quality. This finding underscores that popular content on social media platforms is not always scientifically reliable or of high quality, and that, despite YouTube’s potential as a tool for public health education, its content should be critically evaluated (Canbolat et al., 2024; Kim et al., 2021). In particular, supporting videos produced by healthcare professionals and official institutions that are of substantial duration, reference credible information sources, and contain current and evidence-based content will contribute to enhancing digital health literacy.

### Limitations

This study has several limitations. As a cross-sectional investigation, it captures data from a specific time frame on the continuously evolving YouTube platform; therefore, the findings may diminish in validity over time. The analysis was restricted to the first 200 videos retrieved for each keyword, and other social media platforms, such as TikTok, Instagram, Twitter and Facebook, were not included in the evaluation. Existing literature includes studies that provide comparative analyses of content across various social media platforms and the materials disseminated within them (Denniss et al , 2024; Elkin, Pullon & Stubbe, 2020; Liu et al., 2025; Massey et al., 2016; Saban Bozan, Beşparmak & Küçüksüllü, 2025; Tam et al., 2025). In the literature, there is also a study that evaluated the content of TikTok videos focusing on HPV-related oropharyngeal cancer and the HPV vaccine. In this study, conducted by Matthews et al. (2025), 44% of the videos were created by healthcare professionals, and 72% presented pro-vaccination content; these findings show similarities to the results of our study.

In addition, Zhou et al. (2025) conducted a recent study comparing the information quality and readability of patient education materials on spinal surgery procedures generated by four different artificial intelligence language models (ChatGPT-4o, ChatGPT-o3 mini, DeepSeek-V3, and DeepSeek-R1). This study demonstrates that current research is not limited to evaluating content across different social media platforms but also paves the way for future investigations into the quality and reliability of health information delivery in various fields.

Moreover, the inclusion of only videos produced in Turkish may be regarded as another limitation. Analyzing content in different languages could increase the international generalizability of the findings. Some studies have reported that English-language videos on YouTube possess higher levels of informational quality and overall content standards compared to their Turkish-language counterparts (Kaşıkcı& Yıldırım, 2021; Yaldiz & Meral Koc, 2025). However, considering that individuals generally prefer to access health information in their native language and that the target audience of the present study consisted of Turkish-speaking users, the inclusion of only Turkish-language videos can be justified in terms of the study’s internal consistency and may be regarded as a reasonable limitation. In the future, multicenter comparative studies with larger sample sizes and involving multiple languages are expected to provide stronger and more generalizable contributions to the literature.

Additionally, since November 2021, YouTube has removed public access to dislike counts; therefore, in the calculation of VPI scores, this value was assumed to be 0. Although some studies have employed software tools to retrieve hidden dislike counts or used alternative VPI calculation methods, this approach was not adopted in our study (Di Spirito et al., 2023; Erdoğan et al., 2024). This may be considered an important limitation of our study. Assuming dislikes = 0 artificially inflates the like ratio, which is a component of VPI. Therefore, it is accurate to say this likely inflates the VPI scores, making the lack of correlation with quality even more noteworthy. This highlights the necessity of updating the VPI calculation formula (The YouTube Team, 2021).

## Conclusions

In conclusion, our study determined that YouTube videos related to HPV generally exhibit moderate information quality and low source reliability, with no direct association found between video popularity and content quality. We believe that developing strategies to reduce misinformation on online platforms and increasing the number of videos produced by healthcare professionals may contribute to reducing vaccine hesitancy.

To the best of our knowledge, no prior study has specifically evaluated Turkish-language videos on HPV and the HPV vaccine. Therefore, this research constitutes an original contribution to the existing body of literature. These findings may inform future digital health literacy interventions.

In particular, promoting the production of videos produced by healthcare professionals and official institutions that are of extended duration, reference credible sources, and provide current information will contribute to enhancing digital health literacy.

Furthermore, due to the removal of public dislike counts on the YouTube platform, there is a need to update the VPI calculation formula.

## Supplemental Information

10.7717/peerj.20828/supp-1 Supplemental Information 1 Quality, reliability and popularity assessment of Turkish YouTube videos on Human papillomavirus and its vaccine: content analysis data

## Additional Information and Declarations

### Data Availability

The following information was supplied regarding data availability: The data is available in the Supplementary File.
